# Single-step ambient-air synthesis of graphene from renewable precursors as electrochemical genosensor

**DOI:** 10.1038/ncomms14217

**Published:** 2017-01-30

**Authors:** Dong Han Seo, Shafique Pineda, Jinghua Fang, Yesim Gozukara, Samuel Yick, Avi Bendavid, Simon Kwai Hung Lam, Adrian T. Murdock, Anthony B. Murphy, Zhao Jun Han, Kostya (Ken) Ostrikov

**Affiliations:** 1 CSIRO Manufacturing, P.O. Box 218, Bradfield Road, Lindfield, New South Wales 2070, Australia; 2 School of Physics, The University of Sydney, Sydney, New South Wales 2006, Australia; 3 School of Mathematical and Physical Sciences, The University of Technology, Sydney, New South Wales 2007, Australia; 4 Institute for Future Environments and Institute for Health and Biomedical Innovation, School of Chemistry, Physics and Mechanical Engineering, Queensland University of Technology, Brisbane, Queensland 4000, Australia

## Abstract

Thermal chemical vapour deposition techniques for graphene fabrication, while promising, are thus far limited by resource-consuming and energy-intensive principles. In particular, purified gases and extensive vacuum processing are necessary for creating a highly controlled environment, isolated from ambient air, to enable the growth of graphene films. Here we exploit the ambient-air environment to enable the growth of graphene films, without the need for compressed gases. A renewable natural precursor, soybean oil, is transformed into continuous graphene films, composed of single-to-few layers, in a single step. The enabling parameters for controlled synthesis and tailored properties of the graphene film are discussed, and a mechanism for the ambient-air growth is proposed. Furthermore, the functionality of the graphene is demonstrated through direct utilization as an electrode to realize an effective electrochemical genosensor. Our method is applicable to other types of renewable precursors and may open a new avenue for low-cost synthesis of graphene films.

## Abstract (web summary)

Graphene films are commonly produced by thermal chemical vapour deposition, which is capable of producing high-quality films but still limited by factors such as high cost. Here, the authors report the growth of single-to-few-layer continuous graphene films under ambient-air conditions.

Widely adopted techniques for the synthesis of large-area, homogeneous and highly crystalline carbon nanostructures are primarily based on thermal chemical vapour deposition (CVD) methods, in which purified gases (for example, CH_4_, H_2_, Ar) are processed at elevated temperatures (typically around 1,000 °C) over a prolonged period^1^,^2^. The use of purified gases, while critical to providing a controlled environment for generating the building units necessary for carbon nanostructure growth, is however, expensive, hazardous and requires extensive vacuum processing. Moreover, complex and prolonged processes in the high-temperature environments incur additional operating costs, which further impede the scalability and commercialization of crystalline carbon nanostructures^3^,^4^.

Graphene, an atomically thin film of crystalline carbon, is a highly promising nano-carbon material whose production is subject to the aforementioned limitations. Graphene films hold strong potential for application in diverse technologies, including water filtration and purification, renewable energy, sensors, personalized healthcare and medicine^5^,^6^,^7^. However, efficient, scalable and low-cost production of graphene film with tuneable properties are essential for such technologies to be feasible. This ability remains a critical challenge.

Recent investigations have demonstrated significant progress in addressing several of these concerns to facilitate the translation of graphene technologies into commercial applications. This includes the transformation of carbon precursors of heterogeneous chemical states into graphene-related materials^8^,^9^. In particular, carbon-containing liquid or solid biomass precursors are attractive due to their low cost^9^,^10^. Nevertheless, highly purified carrier gases and lengthy vacuum operations are still required for these precursors. The hazardous nature and high cost of these gases often reduce the production efficiency. It is thus highly topical and important to develop a technologically and environmentally sustainable process that is free of compressed gases for the production of functional graphene films.

Here we present a single-step, rapid thermal synthesis of uniform and continuous graphene films in an ambient-air environment, using a cheap and renewable form of biomass, soybean oil, as the precursor. To the best of our knowledge, this is the first time that the synthesis of graphene film has been demonstrated in an ambient-air environment without any compressed gases. Graphene derived from this unique ambient-air process exhibits good and tuneable film properties, which are comparable to those of graphene synthesized with conventional methods^2^,^11^. This ambient-air process for graphene fabrication is fast, simple, safe, potentially scalable and integration-friendly. Importantly, it offers the scope to potentially address the critical roadblocks towards large-scale, efficient graphene manufacturing.

## Results

### Controlled synthesis of graphene in ambient-air environment

Currently, graphene synthesis involves several key factors need to be improved: (i) lengthy high-temperature annealing processes to increase the grain size of the metal catalyst used to form graphene; (ii) utilization of purified and compressed gases to offer a homogenous and controlled delivery of carbon source materials; and (iii) the use of lengthy vacuum operation to avoid the presence of any detrimental reactive oxygen species from air^2^,^4^. To overcome these problems, we have designed a thermal CVD process to produce graphene in an ambient-air environment that is completely free of compressed or purified gases and requires minimum processing time.

**Figure 1:**
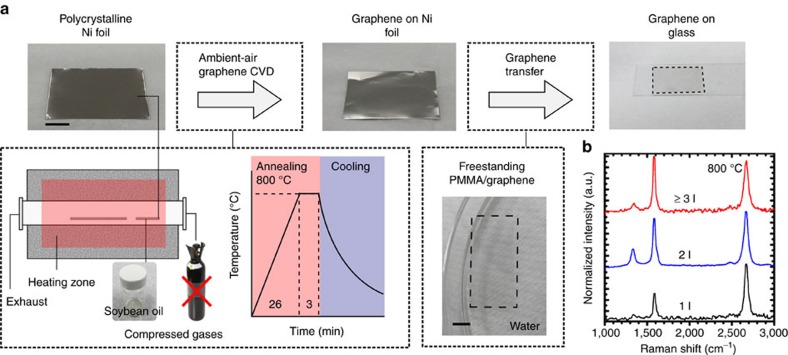
Growing graphene films in the ambient-air process. (**a**) Polycrystalline Ni foil is thermally annealed together with soybean oil precursor, and the controlled synthesis of graphene is promoted in an ambient-air environment. Graphene films can then be transferred onto glass substrate. (**b**) Raman spectra indicate the presence of 1-layer, 2-layer and ≥3-layer regions in the graphene film grown at 800 °C. Scale bar, 1 cm in **a**.

The process is schematically illustrated in Fig. 1a, in which the precursor for graphene growth and a metal catalyst (for example, Ni foil) are placed close together inside the heating zone of a furnace, before heating the quartz tube. The quartz tube is then sealed and the temperature is increased. During the ramping stage, air inside the quartz tube is released through a valve to maintain atmospheric pressure. Once the annealing stage is complete, the sample is removed from the heating zone for rapid cooling. Raman spectra of the samples grown at 800 °C in the ambient-air process indicated the presence of single-to-few layer graphene films covering the surface of the growth substrate (Fig. 1b).

In the standard operation, the catalyst is low-cost polycrystalline Ni foil. Graphene growth occurs by thermal reforming of a natural precursor, soybean oil, in a closed ambient-air environment. Unlike conventional CVD methods or conventional natural precursor methods for growing graphene, the technique does not require any purified gases^8^,^9^. Moreover, expensive vacuum processing is avoided. The natural precursors substituted for purified gases are cheaper and safer. By restricting the air flow into the quartz tube, the transformation of solid-state carbon into carbon dioxide or other gaseous species is prevented. By controlling the temperature, cooling rate and precursor amount, the process enables the growth of homogenous graphene films of good quality. A comparison of the method with other CVD processes is provided in Supplementary Tables 1 and 2.

The parameters observed to control the quality of graphene include temperature, processing time, precursor, substrate and the ambient-air environment. Nickel acted as a good catalyst for the breakdown of precursor material (in this case, the soybean-oil molecules) into smaller building units that are essential for the synthesis of graphene^12^.

To investigate how the transformation occurred in the process, we have analysed the chemical composition of the annealed soybean oils at different temperatures (Supplementary Fig. 1). During the early stages of the annealing process, for instance at 300 °C, the long carbon chains in the soybean oil precursor were thermally dissociated into gaseous carbon building units such as methyl and ethyl species (Supplementary Fig. 1a). Other gaseous species were also generated, including hydrogen, water, hydroxyls and carbon dioxide, as confirmed by mass spectrometry (Supplementary Fig. 1b and c). Traces of heavier hydrocarbons such as propane were also observed. Most of the oil was vapourized by about 425 °C and a rapid mass reduction of the oil was observed by thermogravimetric analysis below 500 °C (Supplementary Fig. 1d). These building units present in the vapour can diffuse through the tube during the heating stage. As the temperature gradually increases to 800 °C, these carbon building units begin to dissociate into carbon atoms and dissolve into the Ni bulk. The sample was annealed for 3 min at 800 °C to promote dissolution of carbon atoms in the Ni substrate. Finally, following the rapid cooling stage, carbon segregates from the bulk and crystallizes on the Ni surface forming graphene^12^,^13^.

At elevated temperatures, long hydrocarbons in the oil decompose in the presence of O_2_ to form water vapour. In particular, water vapour can promote the etching of amorphous carbon deposits on the Ni surface^14^. As such, we did not observe the formation of amorphous carbons in our sample. This also helps maintain the catalytic activity of the Ni surface in breaking down the precursor material^15^. Moreover, we have conducted a detailed analysis on the consumption of oxygen in the reactor during the growth process (Supplementary Note 1). We found that the precursor amount was critical for the consumption of reactive oxygen species. In the optimal growth condition, a slight carbon excessive environment is used to promote the growth of graphene and deter the formation of amorphous carbon. On the other hand, an over-excessive amount of precursor material led to an oversaturation of deposited carbon in the bulk of Ni, and subsequently, the crystallization of graphite on the Ni surface. This may explain the resulting formation of thick graphene sheets as observed in Supplementary Fig. 2a. Moreover, in the case of an insufficient amount of precursor, oxygen species can be present in the as-grown product in the form of C–O amorphous carbons (Supplementary Fig. 2b), consistent with the aforementioned calculations of oxygen consumption (Supplementary Note 1)^16^. These experiments indicate the critical role of the thermally dissociated precursor materials (that is, hydrocarbons) in consuming the reactive oxygen species present in the ambient-air environment, which has a profound effect in controlling the quality of the as-grown graphene films.

We have also noticed that a slow cooling can promote excessive carbon segregation from the Ni bulk, which may account for the observed formation of a graphite-like film (Supplementary Fig. 2c). Another parameter that significantly influences the growth of graphene in the ambient air environment is the annealing temperature. At an annealing temperature of 500 °C, an incomplete formation of the graphene film was observed (Supplementary Fig. 2d). This may be attributed to an insufficient amount of energy to dissociate and reform the precursor material (that is, hydrocarbon species) required for graphene formation. Conversely, at a higher annealing temperature of 900 °C, thicker graphene sheets were observed (Supplementary Fig. 2e). This may arise from the increased rate of carbon diffusion, segregation and graphitization as a result of the elevated temperature. Importantly, these parameters allow us to obtain graphene films with tuneable average thickness and optical transmission, as characterized by Raman spectroscopy and optical transmission (Supplementary Fig. 3).

It is worth mentioning that graphene did not form on other growth substrate materials with significantly lower carbon solubility than Ni, such as the commonly used Cu foil. Moreover, we did not observe graphene formation on graphitic surfaces such as woven carbon cloth (Supplementary Fig. 4). This suggests that the use of Ni (through, for example, carbon solubility, carbon segregation ability, catalytic effect, possibility of formation of oxide in air) and its interaction with the precursor material play a critical role in enabling the growth of graphene films. We also investigated the possibility of transforming other types of renewable oil groups. In particular, we were able to demonstrate the ambient-air growth of similar graphene films from other types of triglyceride (carbon)-containing precursors such as butter (Supplementary Fig. 5). As such, this method is versatile and may be tailored to transform other renewable carbon-containing natural precursors into graphene films.

### Structure and properties of the graphene films

**Figure 2:**
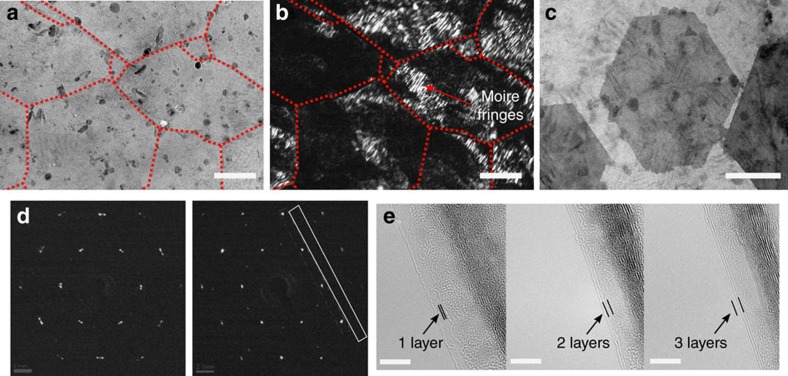
TEM characterization of the graphene film grown in an ambient-air environment. (**a**) Bright-field TEM and (**b**) corresponding dark-field TEM with grain boundaries outlined in red. (**c**) TEM image of mis-oriented hexagonal graphene adlayers and (**d**) selected-area electron diffraction (SAED) patterns. The intensity profile taken from the region outlined by the white box is shown in Supplementary Fig. 6c. (**e**) HRTEM of few-layered graphene films, with the dark regions corresponding to folded edges in the film. Scale bars, 200 nm in **a**–**c** and 5 nm in **e**.

The structural morphology of the graphene film was analysed by transmission electron microscopy (TEM; Fig. 2). The distribution of domain sizes, domain orientations and thickness within the graphene film were characterized. The energy-filtered bright-field and dark-field images were obtained on multiple regions. In the bright-field image, the graphene film appeared uniform, with dark lines representing the overlapping at the grain boundaries (Fig. 2a and Supplementary Fig. 6a). In the dark-field image, the grain boundaries and rotated polycrystalline domains are clearly observed (Fig. 2b and Supplementary Fig. 6b), as indicated by the contrast variations. In addition, the observed Moire fringes (periodic stripes) arise from the mis-oriented overlapping multilayers. This confirmed that the strips/lines of darker contrast were indeed boundaries, as also indicated by the size and shape of the graphene domains. Further, mis-oriented hexagonal graphene adlayers are observed in Fig. 2c. From these TEM characterizations, we can deduce that the graphene film is composed of domains spanning ∼200–500 nm.

In addition, selected-area electron diffraction patterns were taken across a typical region of the sample, where a slight rotation between these patterns was observed (Fig. 2d and Supplementary Fig. 6c). The high-resolution TEM images at the domain edges illustrate the presence of few-layered graphene domains within the film (Fig. 2e). These results demonstrate that the graphene film is composed of mis-oriented domains of turbostratic bi/few-layer graphene^17^.

**Figure 3:**
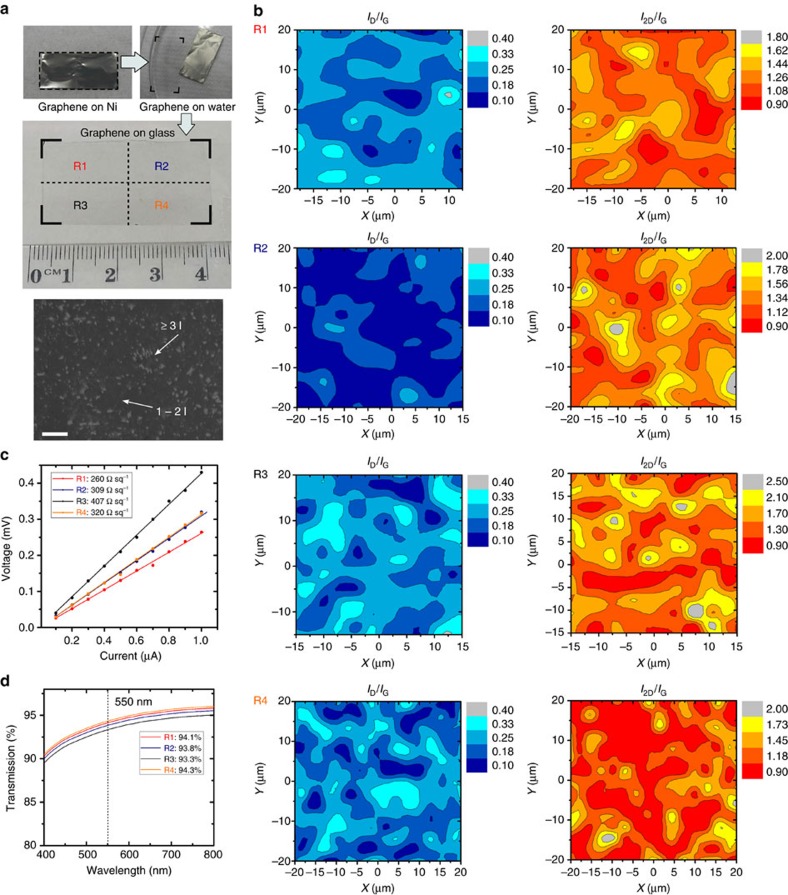
Characterizations of the graphene film grown in an ambient-air environment. (**a**) A 4 × 2 cm^2^ graphene grown on Ni foil is transferred onto glass. Measurements were taken over four quadrants as labelled on the graphene surface. An optical micrograph of the graphene film transferred onto glass is also included. (**b**) Raman spectral analyses of the intensity ratios of *I*_D_*/I*_G_ and *I*_2D_*/I*_G_. (**c**) Four-point probe and (**d**) optical transmission measurements of sheet resistance of graphene films in the respective regions. Scale bar, 20 μm in **a**.

The structural, optical and electrical properties of the graphene film were also analysed by Raman spectroscopy mapping, optical transmission spectroscopy and four-point probe measurements. Before performing these characterizations, a 4 × 2 cm^2^ graphene film grown in the ambient-air process was transferred from the Ni foil substrate to a glass surface, as demonstrated in Fig. 3a. An optical micrograph of the transferred graphene film is also included. The graphene film was observed to grow continuously over the entire surface, with regions of varying thickness (Fig. 3a). To check the uniformity of graphene film, Raman spectral mapping of *I*_D_*/I*_G_ and *I*_2D_*/I*_G_ intensity ratios were taken from four regions R1–R4, as denoted in Fig. 3a. Typically, three distinct peaks are present in the Raman spectra of graphene, namely, the characteristic disorder peak (D-band) at ∼1,350 cm^−1^, the graphitic peak (G-band) at ∼1,580 cm^−1^, and the second-order 2D-band at ∼2,670 cm^−1^. The D-band is attributed to the finite crystallite size effect and various defects induced in the *sp*^2^ carbon materials; the G-band arises from the in-plane vibrational E_2g_ mode of the *sp*^2^-hybridized carbon; and the 2D-band is a second-order Raman spectral feature due to the three-dimensional interplanar stacking of hexagonal carbon networks^18^. For the present film, the intensity ratios of *I*_D_*/I*_G_ is 0.15–0.25 and that of *I*_2D_*/I*_G_ is 0.95–1.50, as shown in Fig. 3b. These values suggest that the film is composed of single- to few-layer graphene. Based on the TEM and Raman measurements, a carrier mobility of 500–750 cm^2^ V^−1^ s^−1^ was estimated for the graphene film (see detailed calculation in Supplementary Note 2)^19^,^20^. Figure 3c shows that the graphene film has an average sheet resistance of 324 Ω sq^−1^, which is consistent with the observed graphene thickness and grain size. Further, an average optical transmittance of 93.9% was obtained (Fig. 3d), suggesting a thin film structure with single-to-few-layer graphene^21^. These characterizations are in good agreement with the microscopic structure of the graphene film (that is, domain size, sheet thickness) obtained by TEM.

**Figure 4:**
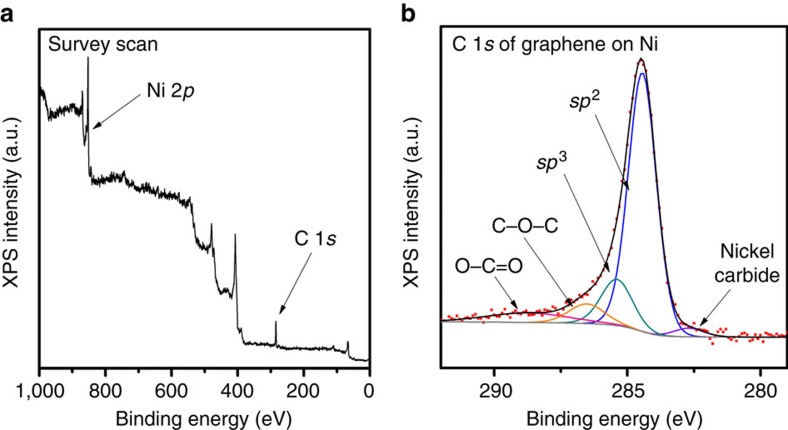
Surface chemical analysis of the graphene film grown in an ambient-air environment. (**a**) XPS survey scan shows the dominant C 1*s* peak, where other peaks are identified from the Ni growth substrate. (**b**) C 1*s* narrow scan and the deconvolution show *sp*^2^, *sp*^3^, nickel carbide and oxygen-attached carbon functional groups.

The surface chemical properties of the graphene film were analysed by X-ray photoelectron spectroscopy (XPS). The survey scan of Fig. 4a shows a dominant narrow C 1*s* peak at the binding energy of 284.5 eV, whereas other peaks were attributed to the Ni growth substrate. The C 1*s* narrow scan in Fig. 4b can be deconvoluted into five peaks, corresponding to the carbon *sp*^2^ (284.5 eV), *sp*^3^ (285.4 eV), nickel carbide (∼282.8 eV) and C–O–C (∼286.5 eV) and O–C=O functional groups (∼288.7 eV; ref. 22). In particular, the graphene film has a good *sp*^2^/*sp*^3^ ratio of ∼5.0, indicating the presence of graphene lattices with good structural quality. These characterizations provide further evidence that the graphene film grown in an ambient-air environment are comparable to those produced by the conventional CVD methods^4^,^11^,^23^.

Moreover, this ambient-air process for graphene synthesis was also applicable to Ni foil growth substrates of lower purity. Graphene films of comparable quality were produced with low-purity polycrystalline Ni foils (99%) rather than high-purity foils (Supplementary Fig. 7). Such low-purity foils offer significant cost reduction in the scale-up for manufacturing graphene films. In addition, there is potential for further scale-up in the production capacity with the utilization of larger reaction chambers.

### Proposed mechanism of graphene growth in ambient-air process

The growth of graphene in an ambient-air environment may initially seem counter-intuitive, as graphene is expected to be destroyed in air at elevated temperatures (above 500 °C). However, we hypothesize that the unique processing conditions promote the controlled synthesis of graphene films in an otherwise destructive environment. Specifically, the thermally dissociated precursor material decomposes in the presence of reactive oxygen species from the ambient-air, leading to the formation of water vapour as a by-product (Supplementary Fig. 1). The water vapour may help suppress the deposition of amorphous carbon, promote the thinning of graphene layers and maintain the catalytic ability of the Ni substrate in breaking down the precursor material into smaller building units necessary for the growth of graphene films.

To better understand the growth process and the possible interaction with Ni substrate, we conducted experiments to probe the surface composition of Ni foils following treatments at elevated temperatures. In particular, we investigated the composition of:

1. Ni foil heat treated in ambient environment without soybean oil, where surface oxidation will be prevalent (Supplementary Fig. 8);
2. Ni foil heat treated in ambient environment with soybean oil, following procedure as outlined previously for the growth of graphene, where surface oxidation may be prevented (Supplementary Fig. 9).

Our XPS analyses showed that when the Ni foil was heated in the ambient environment without soybean oil, oxygen was easily identified on the surface (Ni:O ratio of 1:1.83). However, when the Ni foil was heated with soybean oil, the oxygen content was significantly reduced (Ni:O ratio of 2.69:1). These results indicated that the breakdown of soybean oil in the reaction chamber provided a reaction pathway for the consumption of O_2_, which consequently limited the surface oxidation of Ni at elevated temperatures.

Thus, we propose a growth mechanism based on these supporting evidences. First, soybean oil thermally dissociates into a range of carbon building units, for example, CH_3_, C_2_H_2_ and other species, at the ramping stage (Supplementary Fig. 1). During this stage, molecular fragments of the precursor material may react with and consume O_2_ inside the reaction chamber through possible reaction routes as outlined in Supplementary Note 1. Water vapour produced as a by-product of the consumption of O_2_ may also help suppress the formation of amorphous carbon. The formation of water was supported by the observation of water condensation at the cool ends of the quartz tube outside the heating zone. These molecular fragments may further decompose at higher temperatures to provide a source of carbon dissolved into the Ni foil. This is supported by the detection of an extended nickel carbide peak in the XPS spectra of an etched graphene/Ni sample (Supplementary Fig. 9). Then, growth of graphene can occur through a combination of surface-mediated growth on the Ni foil and precipitation from dissolved species when the sample is cooled. The precipitation step is critical as we observed that the cooling rate was important to control the thickness of the graphene films (Supplementary Fig. 3).

### Graphene as a biosensing electrode

Electrochemical sensing methods for minute amounts of nucleic acid samples offer attractive opportunities for a plethora of preventative health technologies, which require portable, cost-effective and low-power readout devices^24^. In particular, neurodegenerative diseases such as Alzheimer's disease are becoming more prevalent with the ageing population^25^. Alzheimer's disease is best managed with early intervention therapies provided that it can be diagnosed as early as possible. To this end, post-transcriptional epigenetic regulations of gene expressions have been found to provide highly valuable serum-based nucleic acid biomarkers that may be utilized to enable early diagnostic strategies for the disease^26^,^27^,^28^. Consequently, the favourable properties of graphene motivates its applicability as a biosensing electrode.

**Figure 5:**
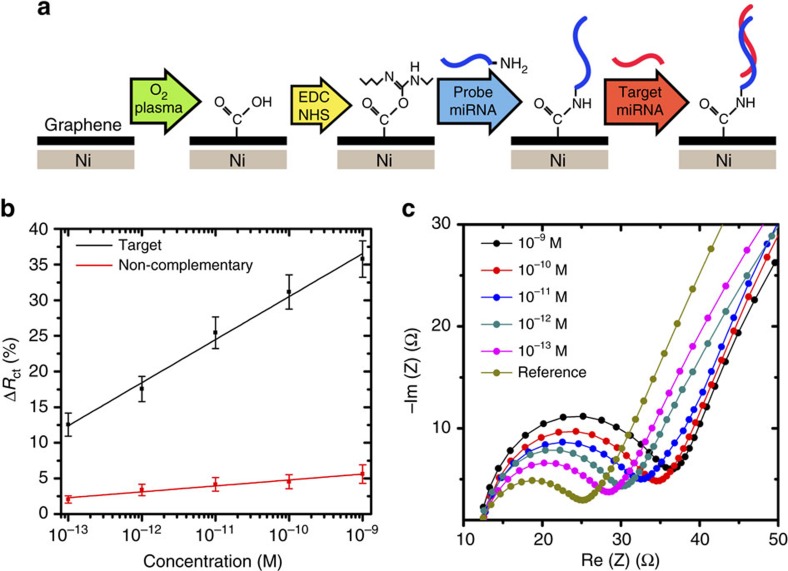
Biosensor assembly and biosensing performance. (**a**) Schematics of the functionalization steps involved for the assembly of the graphene-based electrochemical biosensor. (**b**) Selectivity of biosensor is demonstrated by an increase in *R*_ct_ with increasing concentration of target miRNA. Error bars represent the s.e. of the mean. (**c**) Individual EIS curves showing responses of the biosensor to the target miRNA at different concentrations.

The assembly of the electrochemical graphene-based biosensor is illustrated in Fig. 5a. Briefly, the as-grown graphene was first treated by oxygen plasma to introduce carboxylic functional groups on its surface. Subsequently, carbodiimide chemistry is used to facilitate the covalent immobilization of probe miRNAs, and enable the specific detection of the complementary miRNA sequence (see the ‘Methods' section).

Performance of the graphene sensor was quantified by electrochemical impedance spectroscopy (EIS) technique. The charge-transfer resistance (*R*_ct_) was measured to characterize the response of graphene to the surface immobilization of miRNAs. The *R*_ct_ was observed to increase upon successful immobilization of probe miRNAs on the graphene surface (Supplementary Fig. 10a). With the addition of target miRNAs solution, an increase in Δ*R*_ct_ was observed as the concentration of target miRNAs was increased (Fig. 5b). We define Δ*R*_ct_ by (*R*_ct_−*R*_0_)/*R*_0_, where *R*_0_ is the charge-transfer resistance of the reference sample. This increase in Δ*R*_ct_ is attributed to an impeded charge transport at the graphene surface, caused by spatial blocking of the captured target miRNA molecules. In addition, the hybridization between complementary genomic sequences may induce a build-up of negative surface charge, which may repel negatively charged ferricyanide ions and lead to an increase in *R*_ct_ (ref. 29).

The graphene-based sensor also demonstrates selectivity against miRNA sequences that are mismatched by a single RNA base, as shown in Fig. 5b. A slight increase in *R*_ct_ was observed at elevated concentrations of non-complementary miRNA. This may be attributed to an increase in the non-specifically adsorbed miRNAs. In contrast, the EIS response of graphene in the presence of complementary miRNAs demonstrated a dynamic sensing range spanning 0.1 pM to 1 nM, with a limit of quantification of 8.64 × 10^−14^ M (Fig. 5b,c). Furthermore, the sensing performance of the graphene electrode was evaluated in the presence of common interfering analytes (Supplementary Fig. 10b). The graphene-based sensor demonstrated negligible deviation in *R*_ct_ in the presence of serum albumins and electroactive analytes (that is, uric acid and ascorbic acid) at physiologically relevant concentrations. This suggests that nonspecific binding at the graphene surface did not interfere with the specific binding events with the target miRNA sequence.

The above performance is comparable to other graphene-based electrochemical sensors reported in the recent literature (Supplementary Table 3). For instance, graphene oxide (GO) nanosheets decorated with perylene tetracarboxylic acid diimide have been utilized to enable a detection limit of 5.5 × 10^−13^ M single-stranded (ss)DNA^30^. Similarly, reduced GO have been functionalized with tryptamine to achieve a limit of detection of ssDNA at 5.2 × 10^−13^ M ssDNA^31^; and graphite fibers activated to form GO interfaces were capable of detecting ssDNA down to the concentrations of 5.6 × 10^−12^ M (ref. 32). The graphene film grown in ambient air may thus be promising for future developments of early diagnostic tools, where the quantification of multiple genomic biomarkers in complex biological environments is required.

## Discussion

Graphene films demonstrate excellent functional properties and are promising for diverse applications. However, the high cost and complexities associated with graphene production impede its commercial viability. To this end, we present a novel method for the synthesis of graphene films, in an atmospheric-pressure, compressed-gas-free ambient-air environment utilizing safe, low-cost renewable precursors. This ambient-air method offers numerous advantages over conventional thermal CVD techniques for graphene synthesis, which critically rely on resource- and time-consuming procedures (Supplementary Tables 1 and 2 and Supplementary Note 3). Graphene films with good structural and optoelectronic properties were obtained. On average, the graphene film demonstrated an optical transmission of ∼93.9%, a sheet resistance of ∼324 Ω sq^−1^, Raman *I*_D_*/I*_G_ ratio of 0.15–0.25 and *I*_2D_*/I*_G_ ratio of 0.95–1.50 and domain sizes ranging 200–500 nm. We exemplify the essential process parameters (for example, cooling rate, precursor content, temperature and so on) to enable controlled synthesis and tailored properties of the graphene film in the ambient-air process. Further, we propose a mechanism for the growth of graphene in the ambient-air process, based on depth profiling of the as-grown film, analyses of the ambient-air composition in the reaction chamber and reaction pathways for precursor reforming into graphene. The functionality of the graphene films was demonstrated through its direct integration as an electrochemical genosensor, in which sensitive and selective bio-detection was realized. Importantly, the ambient-air synthesis of graphene films from renewable precursors offers numerous advantages and opportunities for future streamlined integration into large-scale production infrastructures and the realization of diverse graphene-enabled technologies.

## Methods

### Ambient-air thermal synthesis of grapheme

The growth of graphene was carried out in a thermal CVD furnace (OTF-1200X-UL, MTI Corp) with a quartz tube (100 cm in length, 5 cm in diameter). Polycrystalline Ni foils (25 μm, 99.5%, Alfa Aesar) were used as the growth substrate. The experimental schematic is shown in Fig. 1. Briefly, two alumina plates were placed in the heating zone of the furnace. One alumina plate was loaded with 0.14 ml of soybean oil precursor and the other was loaded with the Ni foil growth substrate. The openings of the quartz tube were then sealed. The growth of graphene proceeds with a gradual heating and fast quenching temperature profile. First, the furnace temperature was raised to 800 °C at a rate of 30 °C min^−1^. This was followed by holding at 800 °C for 3 min. After the growth step, the sample was immediately removed from the heating zone to enable a rapid cooling (at approximately 25 °C min^−1^) to segregate the homogeneous and continuous graphene films. Owing to the evaporation and thermal expansion of the precursor material, a small build-up in pressure within the tube was observed. Throughout the heating stage (200 to 800 °C), atmospheric pressure was maintained in the quartz tube by allowing this build-up of gases to exit via the exhaust of the tube. A controlled gas environment was created in the tube through enabling the circulation of gases produced by precursor evaporation. Following the heating stage, pressure within the quartz tube was observed to be stabilized at atmospheric pressure. No additional gases were introduced into the quartz tube throughout the entire growth process.

### Transfer of grapheme

A poly (methyl methacrylate) (PMMA)-assisted transfer of graphene was adopted. Briefly, 46 mg ml^−1^ of PMMA (*M*_w_=996,000; Sigma-Aldrich) was spin-coated onto the as-grown graphene on Ni foil (3,000 r.p.m. for 1 min). The sample was then dried in open air for 12 h. Subsequently, the underlying Ni foil was dissolved in 1 M FeCl_3_ in 30 min. The PMMA/graphene film then floated to the surface. This was washed several times with deionized (DI) water. Next, the PMMA/graphene was lifted off from the DI water bath and transferred onto a glass substrate. The PMMA was then dissolved with acetone, and the sample was repeatedly washed with DI water. The graphene on glass was then used for subsequent microscopy and electrical characterization.

### Microscopy and microanalysis

Raman spectroscopy was performed using a Renishaw inVia spectrometer with Ar laser excitation at 514 nm and a probing spot size of about 1 μm^2^. The XPS spectra were recorded with a Specs SAGE 150 spectroscope with Mg Kα excitation at 1,253.6 eV. Both survey scans and narrow scans of C 1 s and Ni2p_3/2_ were conducted. The Ni and graphene/Ni surfaces were progressively etched through Ar bombardment to create a depth profile of the material. The TEM images were obtained with a JEOL 2200FS TEM microscope operated at 200 kV.

### Optical characterization

Optical images were obtained with an Olympus BX51 optical microscope. Transmittance measurements were obtained using a Varian Cary 5000 ultraviolet–visible spectrophotometer. A graphene area of 4 cm^2^ was used, and optical spectra were recorded in the wavelength range from 300 to 800 nm.

### Electrical four-probe measurements

Silver paint was applied to the graphene transferred onto glass. A graphene area of 1 cm^2^ was used. Four-point probe measurements were conducted at room temperature.

### Inductively coupled plasma mass spectrometry analysis

The Netzsch STA 449 F1 instrument equipped with S-type DTA sensor was used for simultaneous thermogravimetric/differential thermal analysis of the soybean oil precursor samples. Soybean oil samples were placed in Al_2_O_3_ holders, and were heated to required temperatures (300, 500 and 600 °C) at 10 °C min^−1^ heating rate under air purge gas. Correction/blank runs were carried out for each temperature range with empty reference and sample pans before sample thermal analysis. Evolved Gas Analysis was carried out by coupling the Netzsch system to a Thermostar Pfeiffer Quadrupole Mass Spectometry to determine gases and vapours evolving in the atomic mass range of up to 200 a.m.u. (plotted as mass to charge ratio *m*/*z*).

### Biosensor device assembly

The as-grown graphene on Ni foil was treated with a low-temperature O_2_ plasma (100 W, 7 s) to introduce carboxylic functional groups on its surface. The sample was placed flat and 2 cm below the plasma generation zone. The size of each sensing substrate was 2 × 1 cm^2^. Then, the plasma-actived graphene was treated with 0.05 M *N*-(3-dimethylaminopropyl)-*N*′-ethylcarbodiimide hydrochloride (EDC) and 0.03 M *N*-hydroxysulfosuccinimide (NHS) in phosphate-buffered saline (PBS, pH=7, Sigma-Aldrich) for 15 min. This enabled the formation of active ester intermediates via carbodiimide chemistry. Next, the surface of graphene was washed several times with PBS and DI water to remove excess EDC/NHS. Next, the surface of graphene was washed several times with PBS (pH 7, Sigma-Aldrich) and DI water to remove excess EDC and NHS. Then, NH_2_-conjugated miRNAs (probe sequence: 5′-NH_2_-GGTGGAGGGGACGTTTGCAGGT-3′, Sigma-Aldrich) were diluted in PBS to 0.2 μM, and 50 μl was pipetted onto the EDC-treated surface. This was left to incubate overnight in a wet environment and at room temperature. Next, the sensing surface was washed with 0.05% sodium dodecyl sulfonate (Sigma-Aldrich) in 0.04 M hydroxylamine solution (Sigma-Aldrich) to deactivate the remaining carboxylic functional groups and to remove non-specifically bound probe miRNAs. Then, 0.01 M polyethylene glycol (Sigma-Aldrich) was loaded on the sensing surface to block the exposed areas of graphene to reduce further nonspecific binding. Next, the (biomarker) miRNA sequence (target sequence: 5′-CCACCUCCCCUGCAAACGUCCA-3′, Sigma-Aldrich) was dissolved in human serum (Human Plasma AB, Sigma-Aldrich) to obtain dynamic concentrations of 1 nM to 0.1 pM, which were pipetted onto the sensing surface. This was left to incubate at 45 °C for 20 min to induce hybridization between the complementary probe and target sequences. Finally, a washing step with PBS/DI water was used to remove remaining non-specifically bounded target miRNAs. To demonstrate sensing specificity, a similar protocol was adopted by replacing the target sequence with a single-base mismatched miRNA sequence (non-complementary sequence: 5′-CCGCCUCCCCUGCAAACGUCCA-3′, Sigma-Aldrich). This fully assembled device was then utilized in a three-electrode electrochemical cell for biosensing measurements.

### Biosensing measurements

The electrochemical measurements were conducted in 10 mM FeCN_6_ in 0.1 M Na_2_SO_4_ at room temperature. A three-electrode cell configuration was used. The three-electrode cell used the as-grown graphene on Ni as the working electrode, a Pt wire as the counter electrode, and an Ag/AgCl reference electrode. The EIS measurements were conducted in the frequency range from 500 kHz to 1 kHz, using a BioLogic VSP 300 potentiostat/galvanostat instrument. The charge-transfer resistance (*R*_ct_) of the sensing electrode was determined by the diameter of the semi-circle region in the EIS plots. The *R*_ct_ of the sensor following incubation with the target miRNA was expressed as a percentage of *R*_ct_ in the reference (blank) case, which was incubated in the human serum medium in the absence of target miRNAs. To evaluate the contribution of nonspecific interactions, the interfering analytes (5 mM ascorbic acid, 5 mM uric acid and 0.3 μg ml^−1^ BSA, respectively) were diluted in the FeCN_6_/Na_2_SO_4_ electrolyte before the electrochemical measurements. Further, linear regression analysis was utilized to estimate a detection limit for the sensor. From the plot of Δ*R*_ct_ versus concentration, a relation of Δ*R*_ct_=90.89+6.04 log_10_ (Concentration [M]) was deduced (*R*^2^=0.99) for sensing the target sequence. The limit of quantification was calculated by 10*S*_*y*_/*b*, with *S*_*y*_ as the standard deviation of the *y*-intercept (*S*_*y*_=7.24) and *b* as the slope of the linear fit (*b*=6.04; ref. 33).

### Data availability

The data that support the findings of this study are available from the corresponding author on request.

## Additional information

**How to cite this article:** Seo, D. H. *et al*. Single-step ambient-air synthesis of graphene from renewable precursors as electrochemical genosensor. *Nat. Commun.* **8,** 14217 doi: 10.1038/ncomms14217 (2017).

**Publisher's note:** Springer Nature remains neutral with regard to jurisdictional claims in published maps and institutional affiliations.

## Supplementary Material

Supplementary Information Supplementary Figures, Supplementary Tables, Supplementary Notes and Supplementary References
