# Supplementary material for: Single-step ambient-air synthesis of graphene from renewable precursors as electrochemical genosensor
Source: Nat Commun. 2017 Jan 30;8:14217. doi: 10.1038/ncomms14217 (PMC5290271; doi:10.1038/ncomms14217)
Supplement: Supplementary Information — Supplementary Figures, Supplementary Tables, Supplementary Notes and Supplementary References [file ncomms14217-s1.pdf]

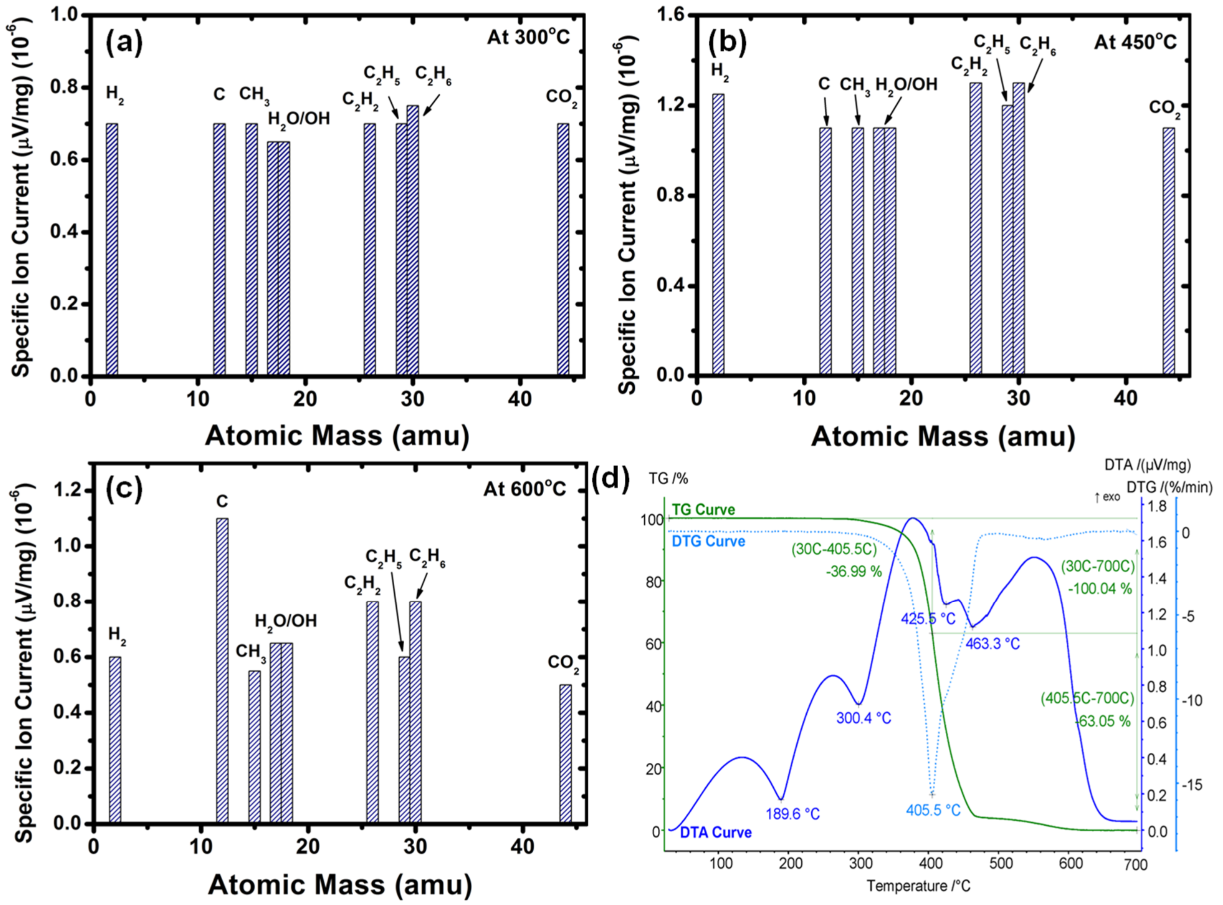

Supplementary Figure 1. Inductively-coupled plasma (ICP) mass spectrometry analyses of vapors produced from soybean oil precursor at different temperatures. (**a**) 300, (**b**) 450 and (**c**) 600 ^{o}C. (**d**) Thermogravimetric analysis (TGA), derivative thermogravimetric analysis (DTG) and differential thermal analysis (DTA) curves of the soybean oil.

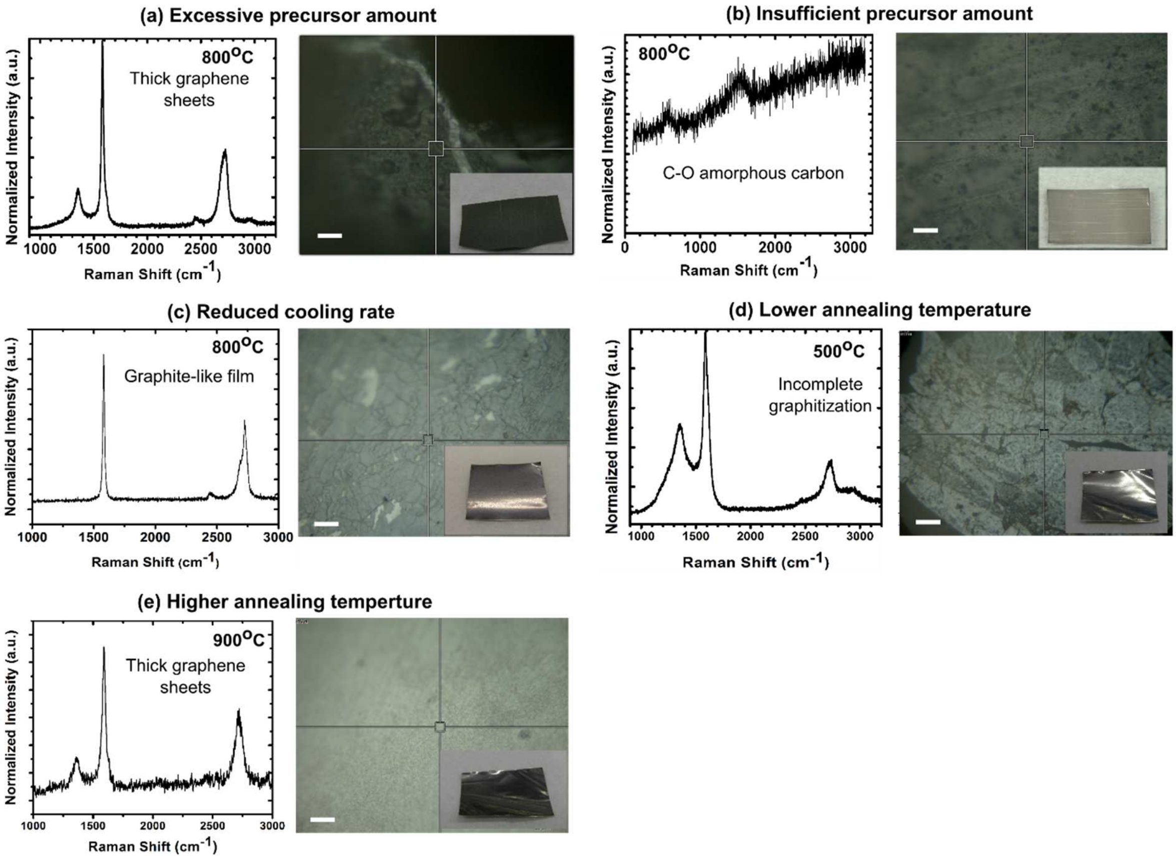

Supplementary Figure 2. Non-optimal growth of graphene thin films and their respective Raman spectra. (**a**) An excessive amount of precursor resulted in the formation of thick graphene sheets; (**b**) an insufficient amount of precursor resulted in the formation of amorphous carbons; (**c**) a slow cooling rate resulted in the formation of graphite-like films; (**d**) a lower annealing temperature (*e.g.,* 500 ^{o}C) led to an incomplete transformation of the precursor; and (**e**) a higher annealing temperature (*e.g.,* 900 ^{o}C) led to thicker graphene sheets. Scale bars: 20 µm in **a**-**e**.

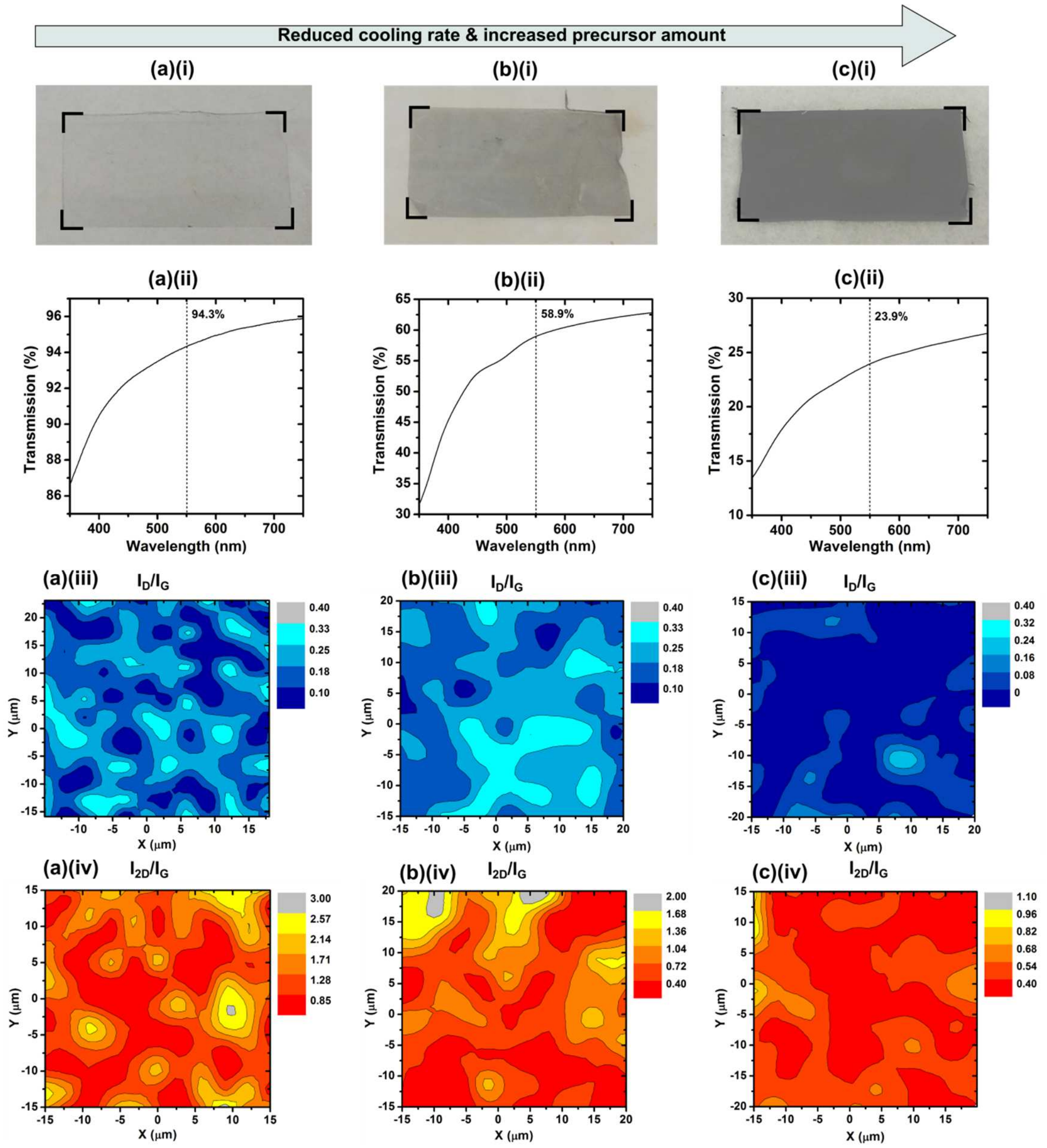

Supplementary Figure 3. Control of graphene film thickness by adjusting the cooling rate and precursor amount in an ambient-air environment. (**a**) Fast cooling rate and an optimal precursor amount, (**b**) slower cooling rate and an increased precursor amount, (**c**) slowest cooling rate and an excessive amount of precursor. (**i**) Optical image, (**ii**) transmission spectra, (**iii**) Raman mapping of I_D/I_G and (**iv**) I_{2D}/I_G measurements of the respective graphene films.

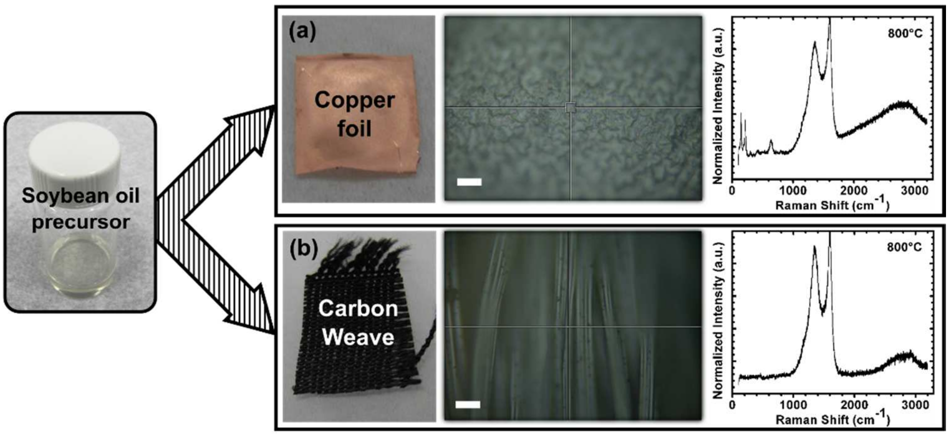

Supplementary Figure 4. Ambient-air process applied to other substrates. Similar growth conditions applied to other substrates of (**a**) copper foil and (**b**) woven carbon. No graphene films were obtained on these substrates. Scale bars: 20 µm in **a**,**b**.

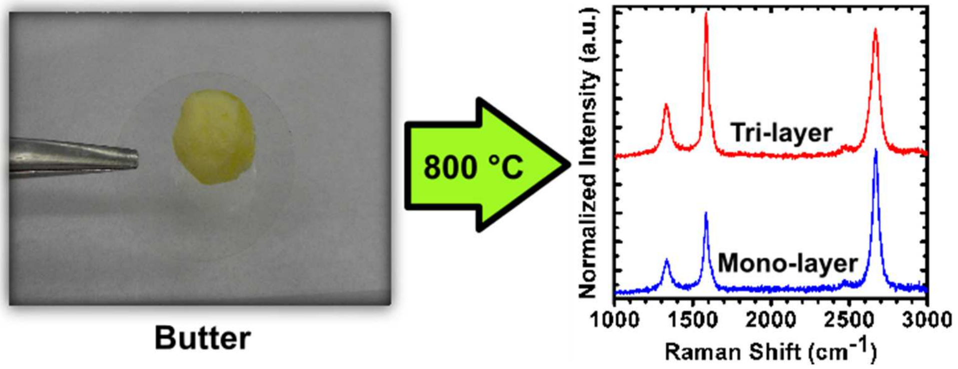

Supplementary Figure 5. Transformation of other fat-containing precursors with the ambient-air process. Butter was used in place of soybean oil, and similar growth conditions were applied. The formation of few-layered graphene films were observed.

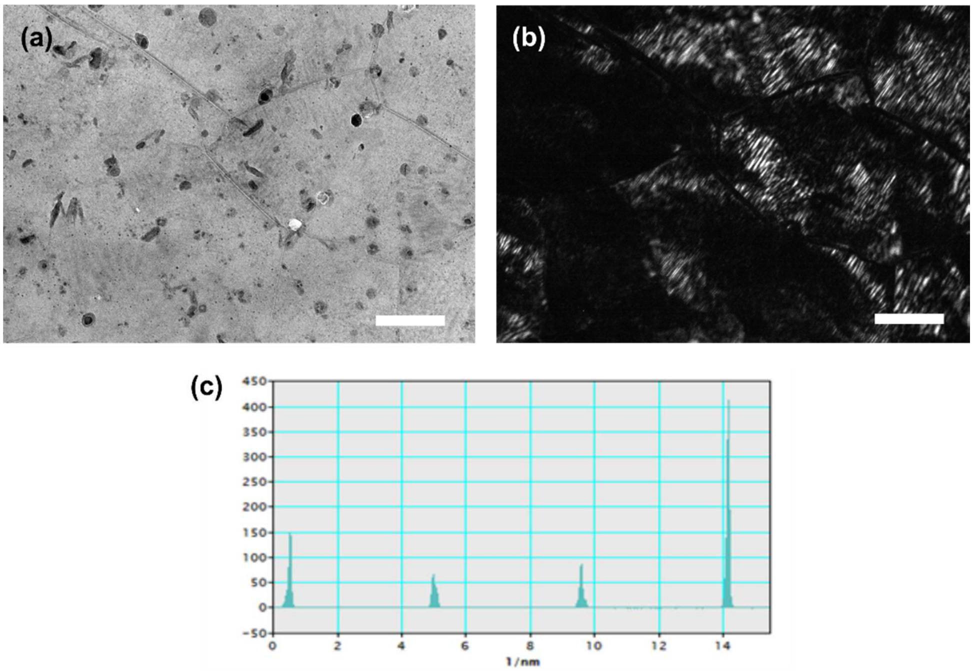

Supplementary Figure 6. Supporting TEM characterizations of the graphene film. (**a**) Bright-field and (**b**) dark-field contrast images of the graphene film, corresponding to Fig. 2a and 2b. (**c**) Respective intensity profile of SAED pattern in Fig. 2d indicating bi/few-layered graphene. Scale bars: 200 nm in **a**,**b**.

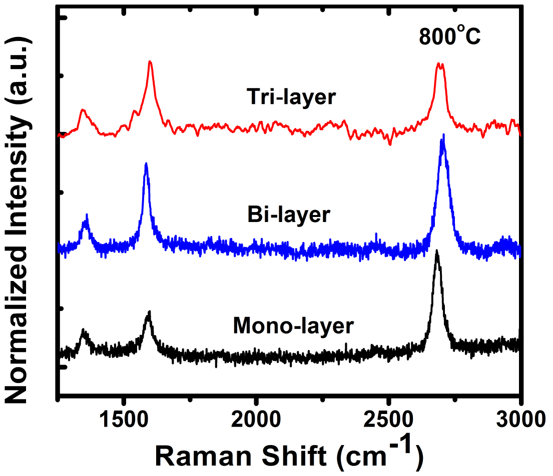

Supplementary Figure 7. Ambient-air process applied to low-purity (99 %) polycrystalline Ni foil growth substrate. Raman spectra indicate the growth of single-to-few layer graphene films at 800 °C.

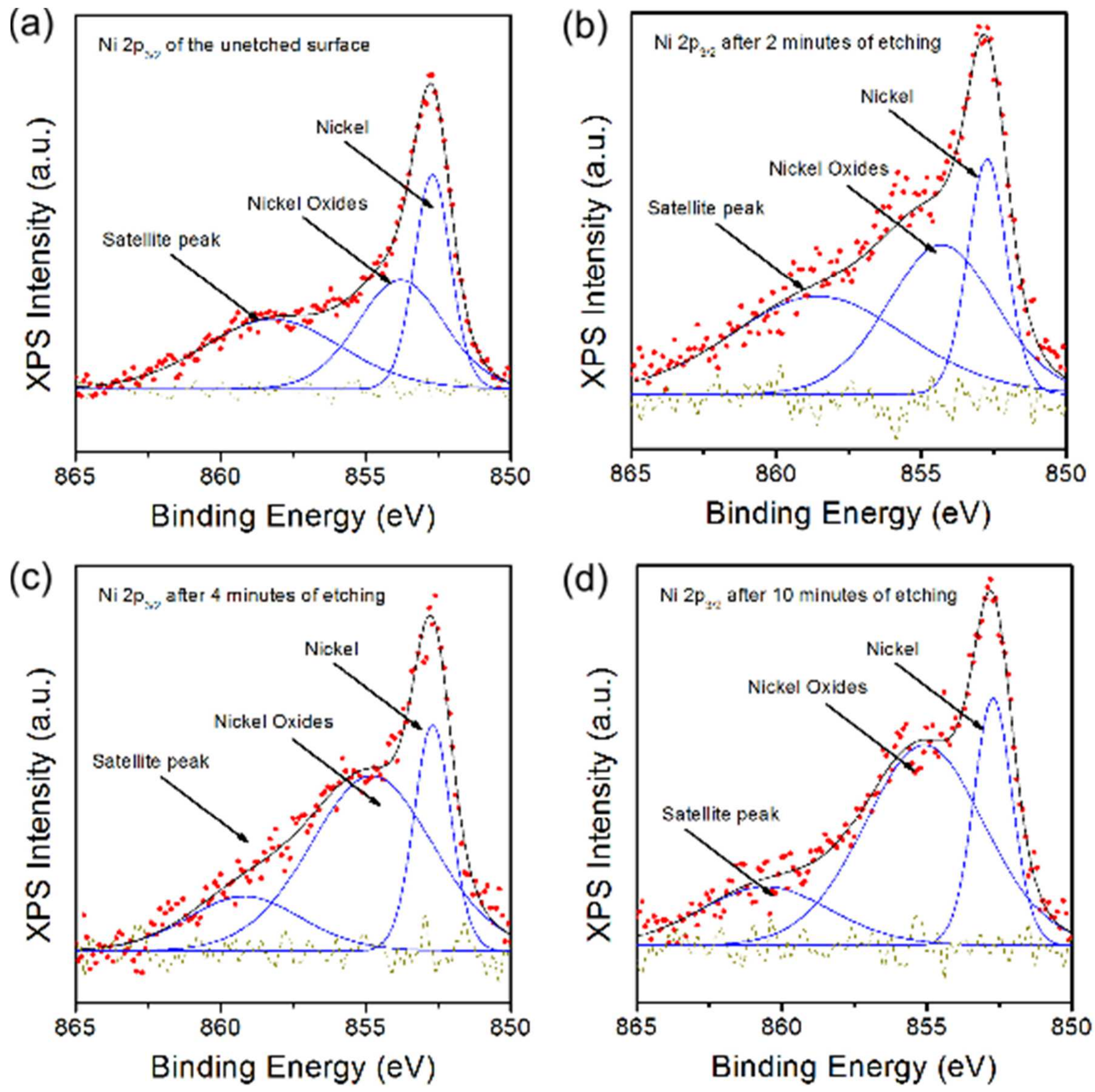

Supplementary Figure 8. Surface analysis of Ni foil thermally heated in the absence of soybean oil. XPS Ni $2p_{3/2}$ spectra of (**a**) Ni surface heat treated without soybean oil and after the etching of (**b**) 2min, (**c**) 4 min and (**d**) 10 min.

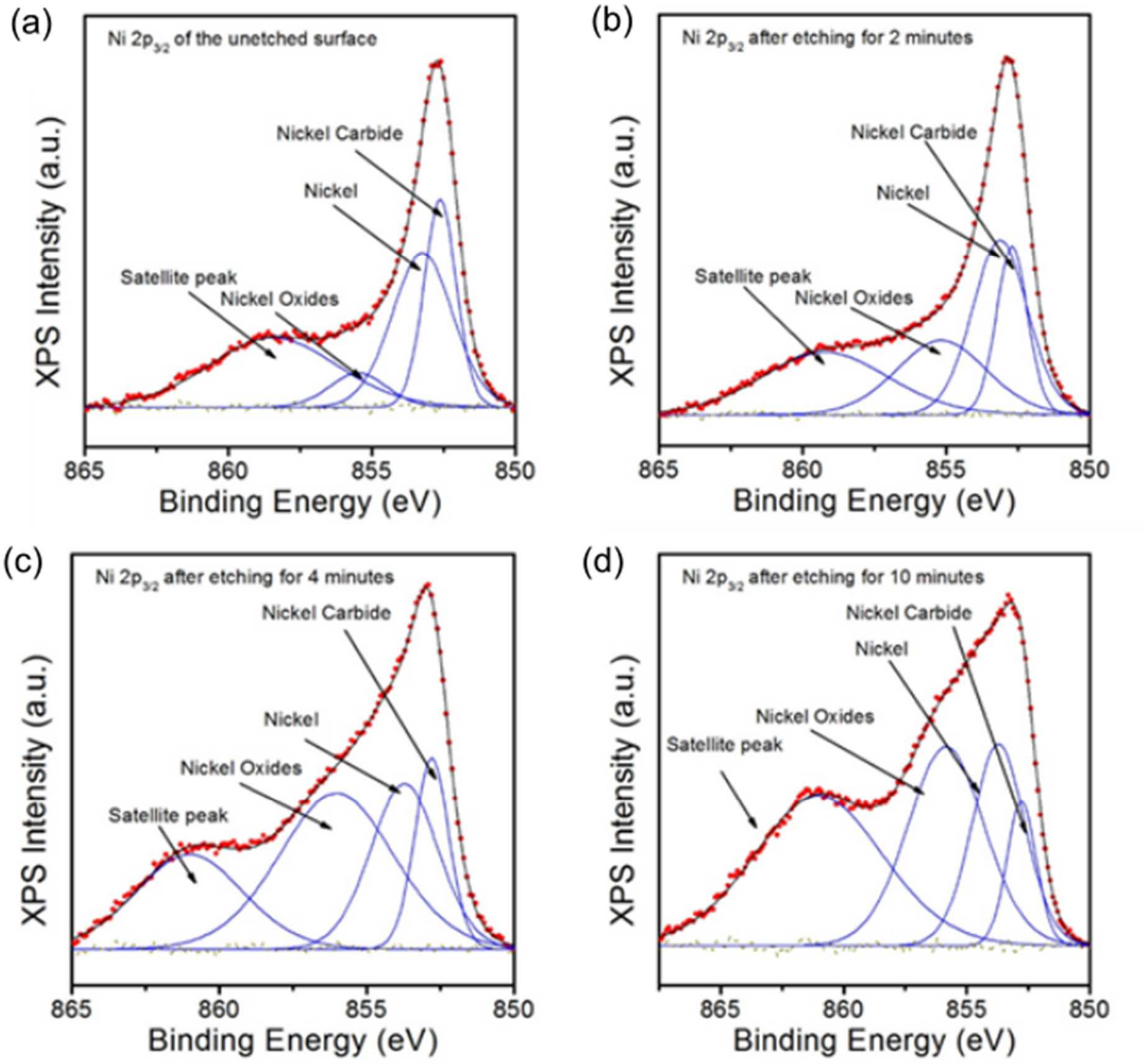

Supplementary Figure 9. Surface analysis of Ni foil thermally heated in the presence of soybean oil. XPS Ni $2p_{3/2}$ spectra of (**a**) graphene/Ni surface and after the etching of (**b**) 2 min, (**c**) 4 min and (**d**) 10 min.

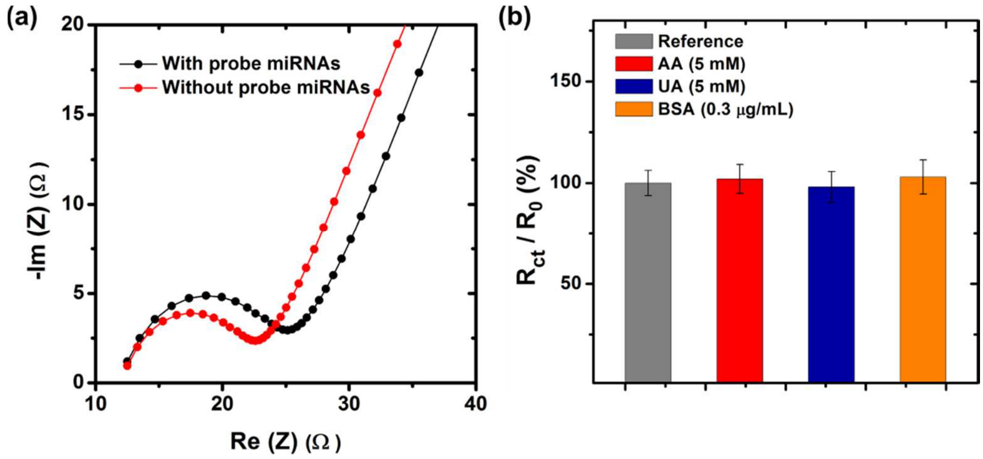

Supplementary Figure 10. Graphene film as a bio-sensing electrode. (**a**) EIS curve showing increase in charge-transfer resistance (R_{ct}) of the graphene electrode upon immobilization of the probe miRNAs. (**b**) Response of the graphene-based biosensor to common interfering analytes, namely, ascorbic acid (AA), uric acid (UA), and BSA (bovine serum albumin), at respective physiological concentrations. Error bars represent the standard error of the mean.

Supplementary Table 1. Comparison of the ambient-air synthesis method with conventional thermal CVD approaches for the production of graphene films.

Metric	Ruoff *et al.* [S1]	Kim *et al.* [S2]	Tour *et al.* [S3]	Bae *et al.* [S4]	This method
Carbon precursor	Methane	Methane	Carbon containing biomasses	Methane	Renewable soybean oil biomass
Feedstock gases	Hydrogen	Hydrogen & Argon	Hydrogen & Argon	Hydrogen	None
Pressure (torr)	0.04 – 0.5	n/a	9.3	0.09 – 0.46	Atmospheric
Synthesis environment	Purified gases	Purified gases	Purified gases	Purified gases	Ambient Air
Processing time (min)	150	140	150	160	< 30
Temperature (°C)	1000	1000	1050	1000	800

Supplementary Table 2. Cost estimate of our method compared to one of the widely-adopted methods for graphene synthesis.

Object of consideration	Our method	Conventional methods†
**Carbon precursor material/ Compressed gases**	Renewable soybean oil biomass<br>\$0.00016 (per run)	Compressed and purified gases<br>\$1.42 (per run)
**Growth substrate**	Ni (25 µm, 99%)\$0.038 (per run)	Cu (25 µm, 99.8%)<br>\$0.015 (per run)
**Electricity for furnace heating**	29 mins in total<br>\$0.33 (per run)	90 mins in total<br>\$1.04 (per run)
**Operation of vacuum pump**	\$0.046 for 26 mins (per run)	\$0.26 for 150 mins (per run)
**Estimated cost (per cm^2)†**	**\$0.40**	**\$2.74**

†Cost estimation in comparison with conventional growth methods adopted from Ruoff and co-workers (Supplementary Reference 1).

Supplementary Table 3. Comparison with graphene-based electrochemical impedimetric biosensors in the recent literature.

**Biosensor**	**Performance (Detection limit)**	**Reference**
Graphene on Ni	8.64×10^{-14} M miRNA	This work
GO with perylene tetracarboxylic acid diimide	5.5×10^{-13} M ssDNA	[S5]
RGO functionalized with tryptamine	5.2×10^{-13} M ssDNA	[S6]
Activated GO/Graphite interface	5.6×10^{-12} M ssDNA	[S7]

Supplementary Note 1. Calculation of oxygen consumption in the reactor during the growth using soybean oil.

(i) Using the dimensions of the quartz tube, the volume of the growth chamber was calculated (0.00196 m^3).

(ii) Providing the dimensions of the Ni foils (4 cm x 2 cm), the surface area of the Ni foils was calculated (as double sided, giving a total of 16 cm^2).

(iii) We clarify as previously provided that the amount of carbon source is 0.14 mL of soybean oil, which is a liquid under ambient conditions.

(iv) Calculations to demonstrate that the amount of solid carbon sources is sufficient to consume all the O_2 in the growth chamber.

In considering consumption of O_2 by the carbon in the growth chamber, we emphasise that this will be a complex process due to the decomposition of soybean oil yielding numerous molecular fragments which consume O_2 through different reaction pathways. This is clear from our results presented in Supplementary Fig. 1 which shows the variety of products (e.g. H_2, C, CH_3, C_2H_2, C_2H_5, C_2H_6 etc.) from the soybean oil precursor at different temperatures, from 300 to 600 °C. The likely combustions reactions include:

$C + O_2 \rightarrow CO_2$	1 C for 1 O_2	-- (1)
$4CH_3 + 7O_2 \rightarrow 4CO_2 + 6H_2O$	1C for 1.75 O_2	-- (2)
$2C_2H_2 + 5O_2 \rightarrow 4CO_2 + 2H_2O$	1 C for 2.5 O_2	-- (3)

$4C_2H_5 + 13O_2 \rightarrow 8CO_2 + 10H_2O$ 1 C for 3.25 O_2 -- (4)

$2C_2H_6 + 7O_2 \rightarrow 4CO_2 + 6H_2O$ 1 C for 3.5 O_2 -- (5)

$2H_2 + O_2 \rightarrow 2H_2O$ solely consumes O_2 -- (6)

Using the growth chamber dimensions and STP conditions, it is calculated that 0.0168 mol $O_{2(g)}$ is present. Also, it is noted that at the temperatures involved in the ambient-air process, CO_2 does not undergo further decomposition.

Using the average density of soybean oil (0.917 g mL^{-1}) and an average chemical composition (linoleic acid - 52%, oleic acid - 25%, palmitic acid - 12%, linolenic acid - 6%, stearic acid - 5%), it is calculated that ~0.0081 mol of C and ~0.0151 mol of H were present in the growth chamber (n.b. an additional ~0.0001 mol of O from soybean oil is also present, which we do not consider further).

If O_2 was only consumed through the reaction of C (reaction (1) above), then O_2 would be slightly in excess with a remainder of 0.0087 mol.

However, all other reaction pathways have a greater consumption rate of O_2. For instance, if O_2 was solely consumed through the reaction of C_2H_5 (reaction (3) above), then all the O_2 will be expended and C will be in excess with a remainder of 0.0035 mol.

We recognise that all these reaction pathways will likely proceed, and so the combined consumption of O_2 will yield an excess of C in the chamber. Furthermore, we noted that the presence of O_2 could be non-uniform in the growth chamber, given that the temperature inside and outside the hot-walled furnace were vastly different. This could lead to the local

environment in the immediate vicinity of the soybean oil precursor and Ni foils to have a significantly lower concentration of O_2. The calculations thus present an upper limit in estimating the amount of O_2 to be consumed by the soybean oil precursor.

We therefore can conclude that the amount of carbon source we use in the experiment- 0.14 mL of soybean oil- is sufficient to consume the O_2 in the growth chamber, yielding an excess of C from which our graphene can form.

Supplementary Note 2. Estimation of carrier mobility for the graphene film.

The carrier mobility of the graphene film is estimated from the defect density in the film, defined by ~$(1/L_a)^2$ [cm^{-2}], in which,

$$L_a = \frac{560}{E_l^4}\left(\frac{I_D}{I_G}\right)^{-1},$$

where L_a [nm] is the crystallite size, E_l [eV] is the excitation laser energy used in the Raman measurements, and I_D/I_G is the Raman intensity ratio of the disorder content. The detailed calculation is shown below.

(i) From Raman characterizations of the graphene film (Fig. 3b in the main text), we deduced an average I_D/I_G ratio of 0.15 – 0.25.

(ii) The Raman measurements were taken with a 514 nm laser. Converting this wavelength to eV, yields excitation energy E_l of 2.41 eV.

(iii) Substituting these variables into L_a, and calculating for the defect density, yields $(1/L_a)^2$ ranging from 8.26×10^9 to 2.27×10^{10} cm^{-2}, respectively, for the lower and upper bounds of the I_D/I_G ratios.

(iv) Consequently, by reference to the work by Hwang *et al.*,[S8] which correlates the defect density to the carrier mobility, we may provide an estimate for our film mobility, in the order of 500 – 750 $cm^2\ V^{-1}\ s^{-1}$. In addition, such mobility is in accordance with Salehi-Khojin *et al.*[S9] and Chen *et al.*,[S10], where a similar morphology, grain size, and defect level in the graphene films were seen.

Supplementary Note 3. Competitive advantages of the present ambient-air graphene synthesis method.

Graphene production inherits high costs and complexities. This impedes its commercial viability. However, this ambient-air technique provides a significantly cheaper, greener, simpler and safer approach for the synthesis of graphene, as compared to the conventional thermal CVD methods (Supplementary Table 1 and Table 2). We attribute this to a key feature unique to this single-step thermal process, the growth of graphene in an ambient-air environment. Consequently, purified gases (*e.g.,* argon, hydrogen, methane) that are expensive and hazardous are not required. Instead, a safe, minimally-processed renewable precursor (soybean oil) functions as the source of carbon, and the ambient-air environment is tailored to enable the growth of graphene films.

In the conventional thermal CVD methods, the processing chamber is firstly evacuated to remove the ambient air. Next, the processing chamber is brought up to atmospheric pressure by filling the processing volume with purified gases. Finally, these purified gases are constantly circulated with extensive vacuum operation over a prolonged duration. These processes maintain an optimal flow of purified gases to enable the growth of graphene.

In the ambient-air process for graphene synthesis, these conventional steps are not necessary. Instead, graphene growth is promoted by direct control of the precursor content, process parameters (*e.g.,* cooling rate, temperature, *etc.*), and ambient-air environment, without the use of any purified gases, in a single-stepped approach. As such, this ambient-air process has the potential to be easily integrated into existing graphene manufacturing infrastructures.

Supplementary References

[S1] Li, X., *et al.* Large-Area Synthesis of High-Quality and Uniform Graphene Films on Copper Foils. *Science* **324**, 1312-1314 (2009).

[S2] Kim, K. S., *et al.* Large-scale pattern growth of graphene films for stretchable transparent electrodes. *Nature* **457**, 706-710 (2009).

[S3] Ruan, G., Sun, Z., Peng, Z. & Tour, J. M. Growth of Graphene from Food, Insects, and Waste. *ACS Nano* **5**, 7601-7607 (2011).

[S4] Bae, S., *et al.* Roll-to-roll production of 30-inch graphene films for transparent electrodes. *Nat. Nano.* **5**, 574-578 (2010).

[S5] Hu, Y., Wang, K., Zhang, Q., Li, F., Wu, T. & Niu, L. Decorated graphene sheets for label-free DNA impedance biosensing. *Biomaterials* **33**, 1097-1106 (2012).

[S6] Zhang, Z., Luo, L., Chen, G., Ding, Y., Deng, D. & Fan, C. Tryptamine functionalized reduced graphene oxide for label-free DNA impedimetric biosensing. *Biosens. Bioelectron.* **60**, 161-166 (2014).

[S7] Zhang, J., *et al.* Scaly Graphene Oxide/Graphite Fiber Hybrid Electrodes for DNA Biosensors. *Adv. Mater. Interfaces* 2, 1-6 (2015).

[S8] Hwang, J. Y., Kuo, C. C., Chen, L. C. & Chen, K. H. Correlating defect density with carrier mobility in large-scaled graphene films: Raman spectral signatures for the estimation of defect density. *Nanotechnology* **21**, 465705 (2010).

[S9] Salehi-Khojin, A.*, et al.* Polycrystalline Graphene Ribbons as Chemiresistors. *Adv. Mater.* **24**, 53-57 (2012).

[S10] Chen, J. H., Cullen, W. G., Jang, C., Fuhrer, M. S. & Williams, E. D. Defect scattering in graphene. *Phys. Rev. Lett.* **102**, 236805 (2009).
